# Evaluation of bone formation following the osteotome sinus floor elevation technique without grafting using cone beam computed tomography: a preliminary study

**DOI:** 10.1186/s40729-019-0181-7

**Published:** 2019-08-01

**Authors:** Panapohn Suk-Arj, Chanchai Wongchuensoontorn, Patrayu Taebunpakul

**Affiliations:** 1 Department of Oral Surgery and Oral Medicine, Faculty of Dentistry, Srinakharinwirot University, Bangkok, Thailand; 2 Department of Oral Surgery and Oral Medicine, Faculty of Dentistry, Srinakharinwirot University, Bangkok, Thailand; 3 Department of Oral Surgery and Oral Medicine, Faculty of Dentistry, Srinakharinwirot University, Bangkok, Thailand

**Keywords:** Sinus lift, Endo-sinus bone gain, Implant protrusion

## Abstract

**Background:**

Osteotome sinus floor elevation (OSFE) is used to increase the bone volume at the site of the maxillary sinus through the transalveolar approach. However, there is uncertainty regarding the necessity of the use of grafting material in order to maintain the space for new bone formation.

**Objective:**

This study aimed to evaluate new bone formation 6 months after osteotome sinus floor elevation without grafting and to evaluate the correlations between residual bone height (RBH), implant protrusion length (IPL), and endo-sinus bone gain (ESBG).

**Material and methods:**

Thirty-one implants (27 patients) from area 14–17 and 24–27 were included in the study. All implants had a history of OSFE without grafting, with cone beam computed tomography (CBCT) taken prior to the surgery. The clinical examination and radiographic examination using CBCT were performed again 6 months after implantation. The RBH, new bone level, ESBG, and IPL were measured. Paired sample *t* test and Pearson correlation were used to analyze the data.

**Results:**

The average RBH before surgery was 7.14 ± 1.07 mm and 6 months after surgery was 8.95 ± 1.17 mm. There was a significant increase in new bone formation in the 6 months following surgery (*p* < 0.05). The average ESBG and IPL were 1.8 ± 0.79 mm and 2.02 ± 0.73 mm, respectively. There was a significant positive correlation between the IPL and ESBG (*p* < 0.05) while there was a negative correlation between RBH and ESBG. This study also demonstrates a decrease in the percentage of bone formation in relation to IPL as the IPL increases. The survival rate of the implant was 100%.

**Conclusion:**

Significant new bone formation can be detected around the implant site 6 months after implantation using OSFE technique without grafting. There is a negative correlation between the RBH and ESBG. While IPL is correlated to ESBG and appears to be the influencing factors of bone formation changes in the maxillary sinus. The preliminary radiographic results suggest that OSFE technique without grafting in combination with optimal IPL can provide sufficient bone height for implant support with a 100% implant survival rate.

**Electronic supplementary material:**

The online version of this article (10.1186/s40729-019-0181-7) contains supplementary material, which is available to authorized users.

## Introduction

Conventional implant placement is often difficult when the residual bone height is inadequate. Support for the implant may be lacking, and there is a risk of perforating the maxillary sinus membrane. In many of these cases, sinus floor elevation is required to increase the bone height to permit successful implant placement [1]. Recent innovations, such as tilting implants and short implants, can be used alongside sinus floor elevation to further improve patient outcomes [2, 3].

Maxillary sinus floor elevation using a lateral window technique is a well-established and effective surgical procedure with a favorable prognosis [1, 4, 5] However, it is more invasive than simple implant placement and is associated with a greater incidence of perioperative and postoperative complications, such as sinus membrane perforation and sinus infection [6, 7]. A less intrusive method of bone augmentation was recently introduced in which the sinus floor is accessed through the alveolar ridge crest with the employment of osteotomes to assist in performing the sinus floor elevation. The osteotome sinus floor elevation (OSFE) technique, introduced by Summers (1994), has proven to be a predictable procedure [8–10].

In cases where there is the insufficient residual bone height (RBH) under the sinus, OSFE is considered to be less invasive and less traumatic [11, 12]. However, there is uncertainty regarding the necessity of the use of grafting material in order to maintain the space for new bone formation. This uncertainty exists because the studies have reported maxillary sinus floor augmentation simply by lifting the sinus membrane without grafting materials [13–15]. This demonstrates that the use of grafting materials is not a prerequisite for predictable bone formation as all achieved successful implant stability and surrounding new bone formation.

Lundgren et al. [16] described a new sinus membrane elevation technique using a lateral approach with a replaceable bone window. This involved elevating the sinus membrane and suturing it to the sinus wall, without the introduction of grafting material. After 6 months of healing in a submerged way, abutments were tightened and the prosthetic steps were undertaken. All implants achieved osseointegration and were stable after 1 year of loading. The authors reported that all implants gained endo-sinus bone, but unfortunately, no measurement of bone gain was provided.

Although a previous study [14] showed the 1-year outcome of 25 implants placed in the resorbed posterior maxilla with an OSFE technique without grafting material, the study showed an endo-sinus bone gain of 2.5 ± 1.2 mm. The study also found that endo-sinus bone gain was significantly correlated with implant protrusion and RBH. Few previous studies focused on the correlation between new bone formation following OSFE and the possible factors that affect this outcome, including the original RBH, and the implant protrusion length. However, most of the previous studies used periapical two-dimensional film for measurement [17, 18]. More recently, cone beam computed tomography (CBCT) has become available and offers a more comprehensive method to view the implant site preoperatively and postoperatively and provides more detailed bone volume and morphology data [19, 20].

This study aims to evaluate new bone formation 6 months after osteotome sinus floor elevation (OSFE) without grafting and to evaluate the correlations between residual bone height (RBH), implant protrusion length, and endo-sinus bone gain. Measurement was taken using CBCT.

## Material and methods

### Study population

A retrospective study of patients who underwent osteotome sinus floor elevation at Srinakharinwirot University or Theptharin Hospital between May 2017 and May 2018 was undertaken. The study was approved by the ethical committee for research in human subjects, Number DENTSWU-EC22/2560 the faculty of Dentistry, Srinakharinwirot University, Thailand. All patients signed an informed consent before being enrolled in the study.

The patient inclusion criteria for this study are as follows: OSFE procedure was performed without grafting material at posterior maxilla region 6 months prior to the start of the study; there was CBCT imaging for assessment of the bone level pre-operation; RBH was ≥ 5 mm at the planned implant site; no history of sinusitis before implant placement; either no underlying disease or the disease is well controlled by the personal doctor; non-smoking patient or smoking cessation at least 1 year; available and detailed implant records, including the implant brand, diameter, length, and the protrusion length; no alcohol addiction; and the use of prosthetic reconstruction using single-implant, single crown restorations that had been fitted within the 4- to 6-month period after implant placement.

The patient exclusion criteria for this study are as follows: patients with sinusitis symptoms and patients who had worn denture on the implant site during the healing period. Twenty-seven patients, with 31 implants, met the enrollment criteria of the study.

CBCT was used to assess bone height and morphology at all implant sites prior to surgery. CBCT was performed again 6 months after surgery. The images were obtained with the WhiteFox® (Acteon Group, Mérignac, France) with the field of view set at 200 × 170 cm and 0.3 mm^3^ voxel size. The preoperative CBCT images were used to determine the treatment protocols for OSFE without grating and simultaneous implant insertion.

### Surgical procedures

Surgical procedure guidelines provided by each of the brand manufacturers (Astra Tech®, Straumann®, Osstem®) were performed under local anesthesia. Thirty-one implants were placed in the posterior region of maxilla using a submerged technique and with one stage surgical procedure. Following a 3–4-month undisturbed healing period, prosthetic treatment for each was undertaken in line with the implant manufacturer’s guidelines.

For pre-operative patient preparation, 1-h prior to surgery, all 27 patients had to orally take an antibiotic drug consisting of 1 g of amoxicillin. Immediately before surgery, all patients rinsed their mouth with 0.2% chlorhexidine solution for 30 s. Local anesthesia was administered in the buccal and palatal regions of the surgical site. Following a mid-crest incision without a releasing incision, a full-thickness mucoperiosteal flap was raised. A round-shaped bur was initially used to mark the implant position. Following a review of the preoperative CBCT scan, minimal pilot drilling (Ø2.2 mm) was performed to a depth approximately 1 mm from the sinus floor. The manufacturer’s guidelines were then followed, gradually increasing the drill diameter until the final diameter drill was used to a depth 1 mm from the sinus floor.

The elevation of the maxillary sinus was developed using the manufacturer’s osteotome instructions at the final drill diameter. Light malleating was performed to achieve the sinus elevation with osteotomies of gradually increasing length until the final depth was achieved. All implants were placed in sites using a submerged technique and using a single-stage procedure.

Standard aftercare treatment was provided following implant placement with the osteotome technique. Rinsing of the mouth with a 0.12% solution of chlorhexidine for 60 s, 2 times a day, for 7 days was prescribed. Anti-inflammatory drugs (ibuprofen 400 mg) 3 times per day were also prescribed with antibiotics (amoxicillin 1 g) to be taken twice daily for 5 days following surgery.

### Prosthetic procedures

To establish if there was continued radiolucency around the implant body, a periapical radiograph for each patient was taken after a healing period of 3–4 months. Dental impressions were then taken and approximately 2 weeks later, prosthetic abutments were inserted and final restorations were completed. Each implant supported a single crown.

### Clinical evaluation

Six months after implant placement, patients were fully informed of the study and signed to confirm their consent to participate. In month 6, the oral examination was performed and CBCT measurements were taken. Any symptoms and complications following surgery regarding sinus infections were recorded

The success criteria proposed by Buser et al. [21] were used including (1) the absence of clinically detectable implant mobility, (2) absence of pain or any subjective sensation, (3) absence of recurrent peri-implant infection, and (4) absence of continuous radiolucency around the implant.

### Analysis of the CBCT imaging

A comparison was made between the preoperative CBCT radiographs and the 6-month postoperative CBCT radiographs. CBCT image analysis was performed with the WhiteFox® imaging software version3.0. The “measure” tool in the software was used for the linear measurement of the bone height between the time intervals analyzed. The radiographic parameters were analyzed by a single dentist on two occasions 1 week apart. The intra-examiner agreement was compared and shown to be good, and the intraclass correlation coefficient (ICC) was 0.94 (*p* ≤ 0.05). All measurements were expressed in millimeters.

In the CBCT images at 6 months after surgery, the center of the measurement tool was positioned at the center of the implant site (Additional file 1: Figure S1). This location in the maxilla was recorded and used as the reference location for the preoperative CBCT images. The maxillary anatomical structures surrounding the implant were referenced when aligning the coronal, sagittal, and axial planes to ensure consistent measurement positions (Additional file 2: Figure S2). Measurements were performed in the coronal and sagittal views of CBCT images. Key radiographic parameters (residual bone height and new bone level) were recorded as previously described [22].

Briefly, the residual bone height (RBH), measured from the preoperative CBCT images showed the vertical distance from the cortex bone under the floor of the maxillary sinus to the alveolar bone crest. The new bone level from the 6-month postoperative CBCT images was measured vertically from the most coronal to most apical of the bone-implant contact area. The vertical bone level at the buccal and palatal sites was measured from the coronal views while those at the mesial and distal sites were determined from the sagittal views of the CBCT images. The mean new bone levels were assessed from the coronal (buccal-palatal) and sagittal (mesial-distal) views of the 31 implant sites at 6 months after the OSFE. The endo-sinus bone gain was then calculated by subtracting the preoperative RBH distance from the postoperative new bone level. A positive outcome would demonstrate a gain of new bone in the sinus.

### Statistical analysis

SPSS software version 20.0 (SPSS Inc., Chicago, IL, USA) was used to analyze all data. Descriptive statistics included the mean and standard deviations (SD) to assess the RBH, new bone level, implant protrusion length, and endo-sinus bone gain. The paired *t* test was used to compare the RBH and new bone level between preoperative and 6 months postoperative CBCT imaging. The Pearson linear correlation coefficient between two independent parameters was calculated for the protruding implant length and the endo-sinus bone gain. One-way ANOVA and post hoc test (Turkey HSD test) were used to compare the differences in endo-sinus bone gain and implant protrusion length between the 3 dental implant systems. The *p* values < 0.05 were considered to be statistically significant**.**

## Results

### Patient characteristics

A retrospective review of patient clinical records from Srinakharinwirot University or Theptharin Hospital between May 2017 and May 2018 identified a total of 27 patients (with 31 implants) who underwent osteotome sinus floor elevation and who had final reconstruction with single-implant crowns by the same surgery technique. They all met the inclusion criteria of the study, including 11 men and 16 women, mean age 54.7 ± 12.1 years (range, 31–77 years).

### Clinical analysis

One patient had vertigo for 2 days following the OSFE and implantation. No other post-operative complications, including sinusitis symptoms, were observed. All implants were clinically stable and fixed prosthesis was fabricated 3 to 4 months after surgery. Abutments and crowns were fitted within the 4- to 6-month period after implant placement. The survival rate for all implants was 100 %.

### Radiographic analysis of new bone formation 6 months following OSFE

**Fig. 1:**
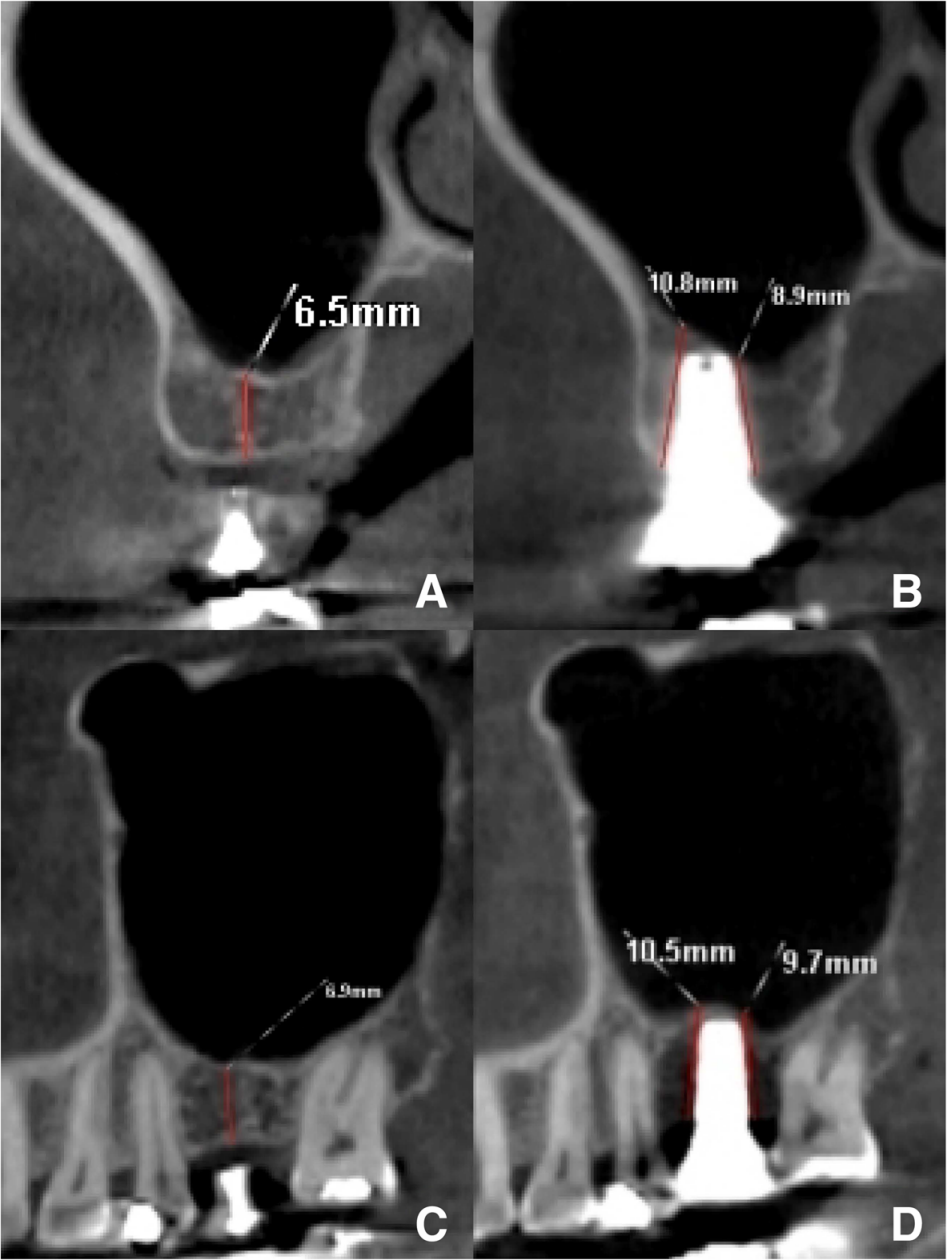
Case 1: The CBCT images of implant site 16 before (left column) and 6 months after surgery (right column). The residual bone height (RBH) was measured from the cortex bone to the alveolar bone crest under the sinus floor in **a** coronal and **c** sagittal views. The new bone level was measured from the most coronal implant thread to the most apical visible implant at **b** buccal and palatal sites in coronal view and **d** mesial and distal sites in sagittal view

**Fig. 2:**
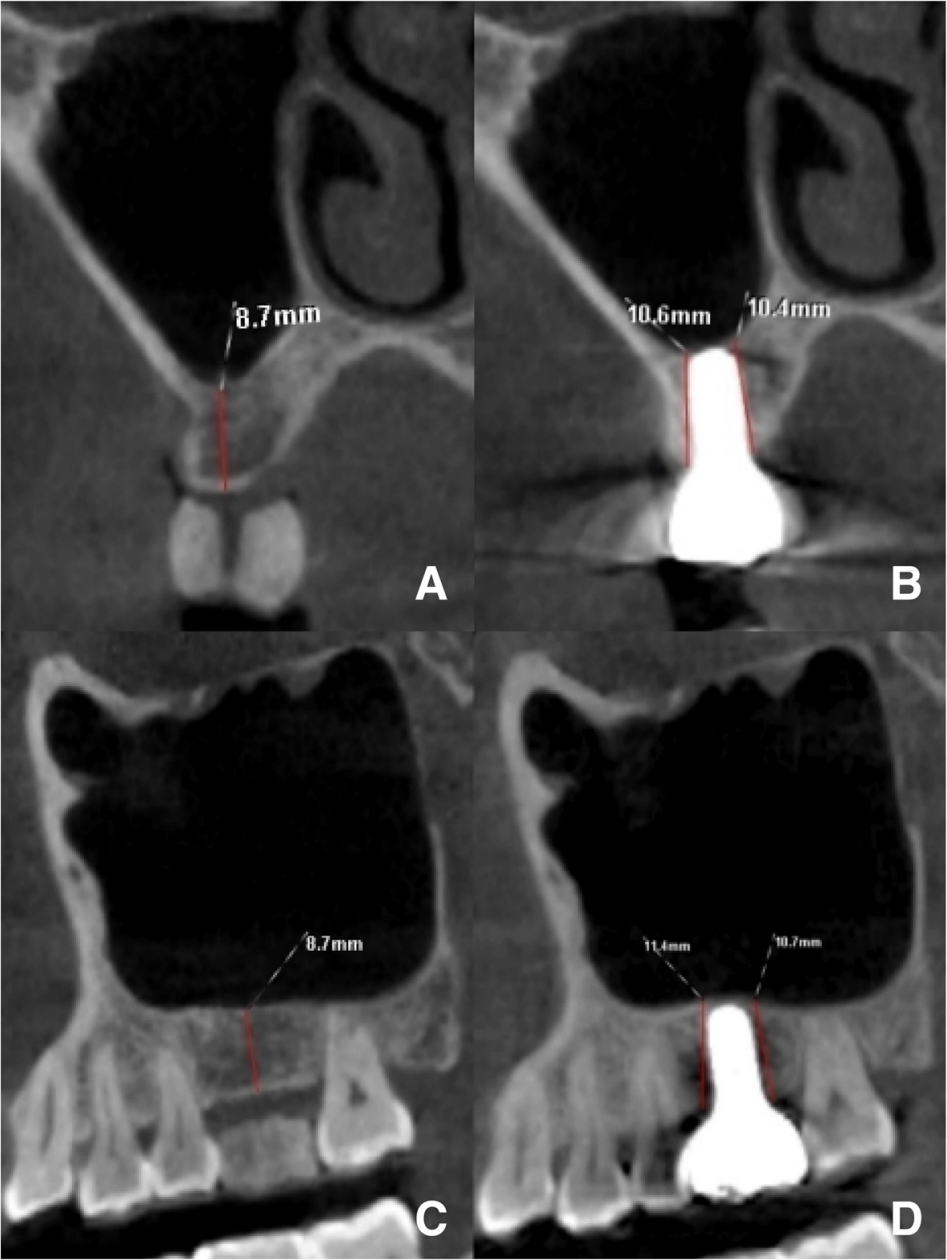
Case 2: The CBCT images of implant site 16 before (left column) and 6 months after surgery (right column). The residual bone height (RBH) was measured from the cortex bone to the alveolar bone crest under the sinus floor in **a** coronal and **c** sagittal views. The new bone level was measured from the most coronal implant thread to the most apical visible implant at **b** buccal and palatal sites in coronal view and **d** mesial and distal sites in sagittal view

The average residual bone height before surgery and the new bone level 6 months after surgery were 7.14 ± 1.07 mm and 8.95 ± 1.17 mm in coronal view and 6.95 ± 0.91 mm and 9.10 ± 1.02 mm in sagittal view, respectively. There was a significant increase in new bone formation after 6 months (*p* < 0.05) (Table 1). The average of endo-sinus bone gain was 1.80 ± 0.79 mm and 1.96 ± 0.67 mm in coronal view and sagittal view, respectively. Figures 1 and 2 showed CBCT images of implant site 16 before (Fig. 1a, c and Fig .2a, c) and after surgery (Fig. 1b, d and Fig. 2b, d) in two different cases.

**Table 1:** Bone levels on CBCT images at preoperative and 6 months postoperative

View	Time	Average bone height (mm)	95% confidence interval of the difference		*p* value
			Lower	Upper	
Coronal view	Preoperative	7.14 ± 1.07	1.50	2.09	*p* < 0.05
	6-month postoperative	8.95 ± 1.17			
Sagittal view	Preoperative	6.95 ± 0.91	1.86	2.44	*p* < 0.05
	6-month postoperative	9.10 ± 1.02

### Radiographic analysis of new bone formation in relation to implant protrusion length (IPL)

The protrusion length for the 31 implants included in the study, ranged from 1 mm to 3 mm with an average of 2.02 ± 0.73 mm. The mean of endo-sinus bone gain was found to be 1.80 ± 0.79 mm and 1.96 ± 0.67 in coronal view and sagittal view, respectively. Pearson correlation analysis showed the implant protrusion length was significantly correlated to endo-sinus bone gain in both views (Table 2).

**Table 2:** Correlation between implant protrusion length and endo-sinus bone gain

View of CBCT	Average implant protrusion length (mm)	Mean of endo-sinus bone gain (mm)	Pearson correlation	*p* value
Coronal view	2.02 ± 0.73	1.80 ± 0.79	0.56	*p* < 0.001
Sagittal view		1.96 ± 0.67	0.53	

**Fig. 3:**
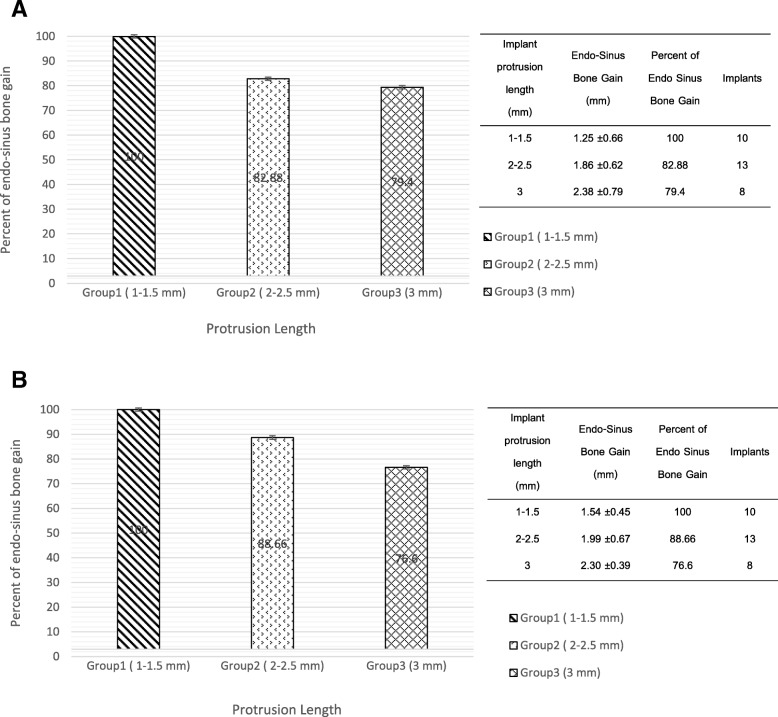
The endo-sinus bone gain in relation to implant protrusion length in **a** the coronal view and **b** sagittal view

The implant protrusion lengths were categorized into 3 groups; group 1, 1–1.5 mm; group 2, 2–2.5 mm; and group 3, 3 mm. There were 1.25 mm, 1.86 mm, and 2.38 mm of endo-sinus bone gain, respectively, equating to 100%, 82.88%, and 79.45% in coronal view (Fig. 3a) and also 100%, 88.66, and 76.6% in sagittal view (Fig. 3b).

### Radiographic analysis of new bone formation in relation to residual bone height (RBH)

The average height of the original bone in coronal view and sagittal view were 7.14 ± 1.07 mm (range 5.3–8.7 mm) and 6.95 ± 0.91 mm (range 5.5–9.0 mm), respectively. There was a negative correlation between RBH and endo-sinus bone gain in both views (Table 3).

**Table 3:** Correlation between the residual bone height and endo-sinus bone gain

View of CBCT	Residual bone height	Endo-sinus bone gain	Pearson correlation	*p* value
Coronal view	7.14 ± 1.07	1.80 ± 0.79	− 0.23	*p* > 0.05
Sagittal view	6.95 ± 0.91	1.96 ± 0.67	− 0.38	*p* < 0.05

### Radiographic analysis of new bone formation in relation to dental implant systems

Of the 31 dental implants, a total of 3 implant systems were used, made up of 7 implants from *Astra* Tech® (OsseoSpeed *TX straight*), *5 implants from Straumann®* (Bone Level SLA straight), and 19 implants from Osstem® (TSIII SA Taper). The implant diameter (range 4.0–5.5 mm) and length (range7.0–10 mm) were properly sized to the residual bone height and bone width.

The heterogeneity among these implant systems was as follows: Both Osstem® and Straumann® systems were SLA (Sand-blasted, Large-grit, Acid-etched) surfaces while Astra tech® was OsseoSpeed (Titanium Oxide Blasted and Acid-etched) surface. There were 2 types of dental implant shape, 12 straight dental implants (Astra Tech® TX and Straumann® Bone Level), and 19 taper dental implants (Osstem® TSIII). In addition, Astra Tech® has MicroThread implant design as the unique feature.

The average protrusion length of the 3 dental implant systems was shown in Table 4. There was no significant difference in the average protrusion length between the groups (*p* > 0.05). The highest endo-sinus bone gain in both coronal and sagittal views was observed in the Astra Tech® system followed by Straumann® and Osstem®. The only significant difference in endo-sinus bone gain between the dental implant systems was observed between Astra Tech® and Osstem® in sagittal view (Table 4).

**Table 4:** New bone formation in relation to the implant system

	Dental implant systems			*p* value
	Astra Tech® (*n* = 7)	Straumann® (*n* = 5)	Osstem® (*n* = 19)	
Average protrusion length (mm)	2.07 ± 0.45	2.20 ± 0.75	1.94 ± 0.83	> 0.05
Endo sinus bone gain (mm)	2.37 ± 0.76(C)	1.83 ± 1.08(C)	1.58 ± 0.64(C)	0.435^a^ 0.788^b^ 0.058^c^
	*2.54 ± 0.71*(S)	1.96 ± 0.60 (S)	*1.74 ± 0.56*(S)	0.231^d^ 0.765^e^ *0.014* ^f^

Italics denote statistical significance

(C) coronal view, (S) sagittal view

^a^No significant difference between Astra Tech® and Straumann® in coronal view

^b^No significant difference between Straumann® and Osstem® in coronal view

^c^No significant difference between Astra Tech® and Osstem® in coronal view

^d^No significant difference between Astra Tech® and Straumann® in sagittal view

^e^No significant difference between Straumann® and Osstem® in sagittal view

^f^Significant difference between Astra Tech® and Osstem® in sagittal view

## Discussion

The present retrospective study evaluated a total of 31 implants placed in 27 patients, following the osteotome sinus floor elevation technique without grafting. The implant survival and success rates of 100% were observed.

This study showed the average of residual bone height before surgery was 7.14 ± 1.07 mm, and 6 months after surgery, the new bone level had increased to 8.95 ± 1.17 mm in coronal view, giving an average of endo-sinus bone gain of 1.80 ± 0.79 mm. The average new bone level after surgery also increased in sagittal view (Table 1). This result confirms the new bone surrounding the implant apex following the OSFE procedure was sufficient to support the implant and increase the bone to implant contact (BIC), which in turn improves long-term survival rates [23]. Cricchio et al. and Si et al. [18, 24] also reported similar results with periapical film measurement.

There is uncertainty regarding the necessity of a grafting material in order to maintain the space for new bone formation. Simultaneous application of grafting materials with a transalveolar sinus floor elevation procedure has been reported to result in improved outcomes [15]. However, Lai et al. [4] reported the application of OSFE in combination with grafting materials did not demonstrate a significantly higher survival rate compared with the non-grafting sites. Gabbert et al. and Nedir et al. [17, 25] also reported high survival rates for implants placed using OSFE without grafting.

In OSFE without grafting procedures, after membrane elevation, the implant protruded through the residual maxillary ridge in the sinus. A blood clot was allowed to form in the submembrane space to act as a scaffold for new bone formation [16]. Previous studies of guided bone regeneration have shown good bone regeneration with the use of blood clots alone, without any use of bone graft [26]. The protruding implant is thus considered to serve as a space-maintaining device under the lifted membrane and to contribute to bone formation around the implant according to the principle of guided tissue regeneration [27]. Blood clots have been shown to contain endogenous growth factors and therefore have the potential to stimulate bone formation [28]. Similarly, a recent experimental study pointed out that the osteoinductive properties of the coagulum are limited primarily by the inability to maintain space under the lifted membrane [5].

Across the 31 implants in this study, the bone formation around the protruding implant has shown considerable variation in volume. This appears to be limited to a formation below the level of the implant apex. No implants showed bone formation coverage of the implant apex.

RBH and implant protrusion length are thought to be the influencing factor of endo-sinus bone gain [29]. In the present study, the average residual bone height and implant protrusion length were 7.14 ± 1.07 mm and 2.02 ± 0.73 mm, respectively. There was a negative correlation between RBH and endo-sinus bone gain, but a significantly positive correlation between IPL and endo-sinus bone gain. This finding was similar to the result of Nedir et al. [14]. These results may be explained by the increased space under the elevated sinus caused by the increased protrusion length. In the present study, it can be seen in Fig. 3 that there is greater bone formation in the group with the longest protrusion. However, the percentage of bone formation in relation to the protrusion length decreases as the protrusion length increases. This may be explained by the variation in pressure created under the sinus membrane by differing lengths of protrusion. The longer protrusions create a greater space under the membrane, causing a greater pressure and therefore greater potential shrinkage of the blood clot, which in turn may reduce bone formation [28, 30]. In an experimental study of sinus augmentation using blood clots alone, Xu et al. [31] reported collapse of blood clots and instability of newly formed bone during the early postoperative stage of healing, and such circumstances can lead to significant reductions in the augmented space and thus insufficient bone formation and poor predictability of implants [32].

Of the 3 dental implant systems used in this study, Astra Tech® showed the highest endo-sinus bone gain in both the coronal view and sagittal view (Table 4). The Astra Tech® system was the only system to use a micro-thread design, and this may explain why it achieved the greatest endo-sinus bone gain. A previous study reported that implants with a micro-thread design promote faster osteogenesis leading to greater bone formation at the implant site. The same study also found micro-thread design provides better primary implant stability, which also promotes new bone formation [33]. In a separate study, micro-thread implants showed a higher significant difference in the percentage of new bone volume and bone to implant contact when compared to macro-thread implants [34]. In two further studies, the two other variations in the implant systems, shape (straight vs taper), and surface (SLA vs OsseoSpeed) showed no significant difference to implant stability or bone formation [35, 36]

Grafting the sites is thought to improve the primary stability by providing more bone onto which the implant can anchor [13, 37]. Moreover, the application of grafting material is thought to create and maintain the space for new bone formation. Some researchers have attempted to investigate the bone remodeling and bone formation under the elevated sinus with simultaneous grafting [11, 38, 39]. However, Pjetursson et al. [1] described a cloudy dome structure with a hazy demarcation observed for grafting sites, which showed signs of shrinkage. The other researchers reported similar observations. Their results were in favor of grafting from the point of view of bone gain measured from panoramic and intra-oral radiographs, but it should be noted that it is difficult to judge from a panoramic or an intra-oral radiograph whether the grafting material has transformed into the bone [38]. Si et al. [24] studied the results of dental implant placed using OSFE with and without simultaneous grafting. They concluded that the application of simultaneous grafting has no significant advantage in terms of clinical success.

This present study also found that the clinical outcomes were excellent and that new bone formation under the elevated sinus floor was evident following implant placement using OSFE without simultaneous grafting. However, this was a limited 6-month retrospective study and prospective studies over a longer time period with increased sample volumes would provide more comprehensive data and in turn, greater certainty in result conclusions.

## Conclusion

The present study focused on the endo-sinus bone gain of implants placed without grafting. New bone formation in the elevated sinus was visible and the endo-sinus bone gain was 1.8 ± 0.79 mm at the 6-month follow-up. Significant new bone formation can be detected around the implant site 6 months after implantation using OSFE technique without grafting. RBH has a negative correlation to endo-sinus bone gain, and implant protrusion length has a significantly positive correlation to endo-sinus bone gain. Implant protrusion length appears to be the influencing factor on bone formation in the maxillary sinus. These preliminary radiographic results suggest that OSFE technique without grafting in combination with optimal implant protrusion length can provide sufficient bone height for implant support with a 100% implant survival rate.

## Additional files

Additional file 1: **Figure S1.** The CBCT images at 6 months after surgery. The center of the measurement tool (intersection point of 2 lines) was positioned at the implant site in (A) axial view (B) coronal view and (C) sagittal view. (TIFF 9050 kb)

Additional file 2: **Figure S2.** The preoperative (right column) and 6-month postoperative (left column) CBCT images in (A, B) axial view, (C, D) coronal view, and (E, F) sagittal view. The location in the maxilla CBCT images at 6-month postoperation (A, C, E) was recorded and used as the reference location for the preoperative CBCT images (B, D, F). The maxillary anatomical structures surrounding the implant were referenced when aligning the coronal, sagittal, and axial planes to ensure consistent measurement positions. When the postoperative images were produced by CBCT, the position of the mandible may not be in the same location as the preoperative CBCT images. Therefore, the locations in the mandible cannot be used as references. (TIFF 7918 kb)

## Data Availability

The datasets used and analyzed during the current study are available from the corresponding author on reasonable request.

## Abbreviations

CBCT: Cone beam computed tomography
ESBG: Endo-sinus bone gain
ICC: Correlation coefficient
IPL: Implant protrusion length
OSFE: Osteotome sinus floor elevation
RBH: Residual bone height
SD: Standard deviations
