# Supplementary figures and images for: Evaluation of bone formation following the osteotome sinus floor elevation technique without grafting using cone beam computed tomography: a preliminary study
Source: Int J Implant Dent. 2019 Aug 1;5:27. doi: 10.1186/s40729-019-0181-7 (PMC6669224; doi:10.1186/s40729-019-0181-7)

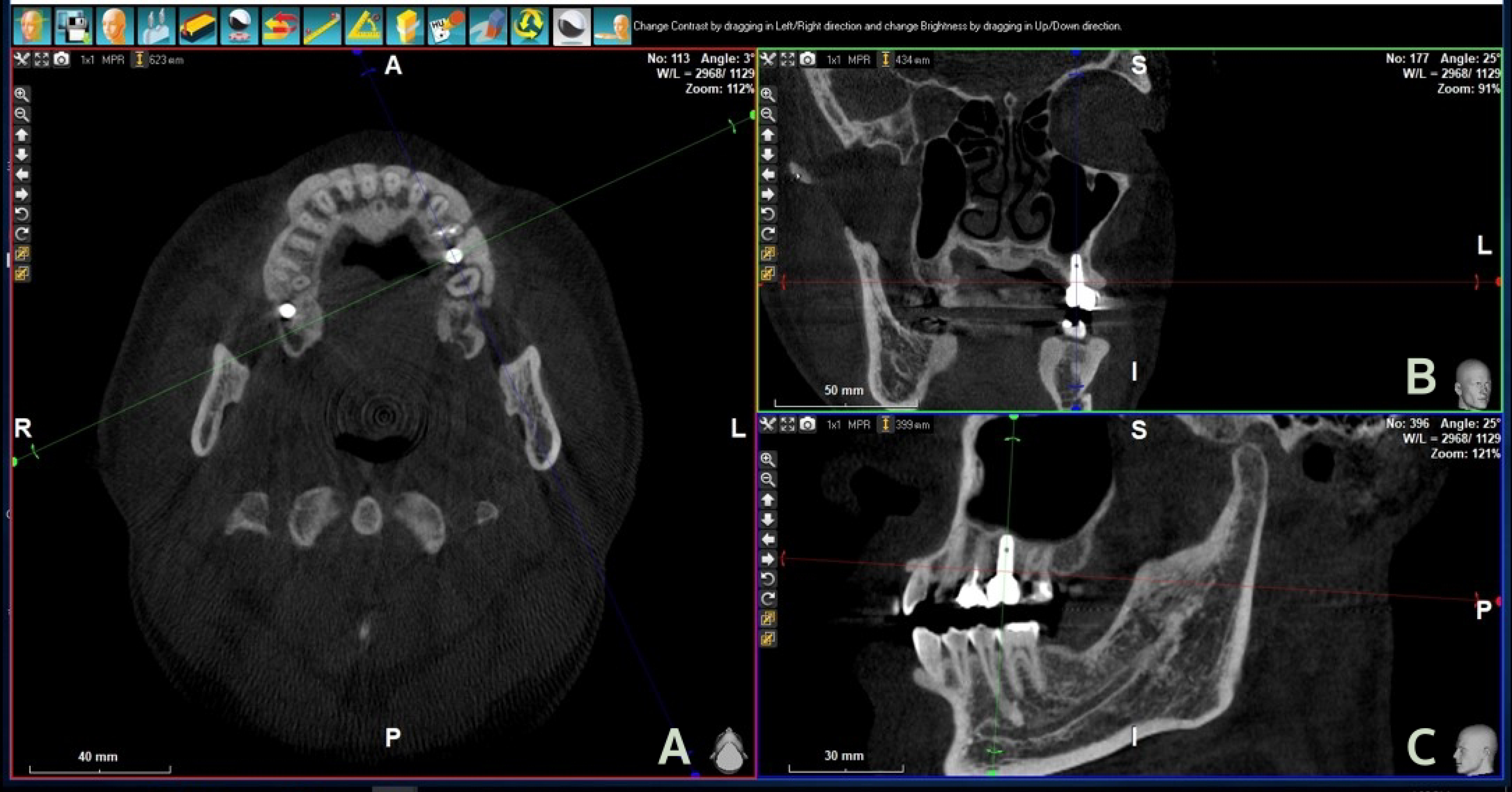

Supplement: Supplementary file 1 — Figure S1. The CBCT images at 6 months after surgery. The center of the measurement tool (intersection point of 2 lines) was positioned at the implant site in (A) axial view (B) coronal view and (C) sagittal view. (TIFF 9050 kb) [file 40729_2019_181_MOESM1_ESM.tiff]

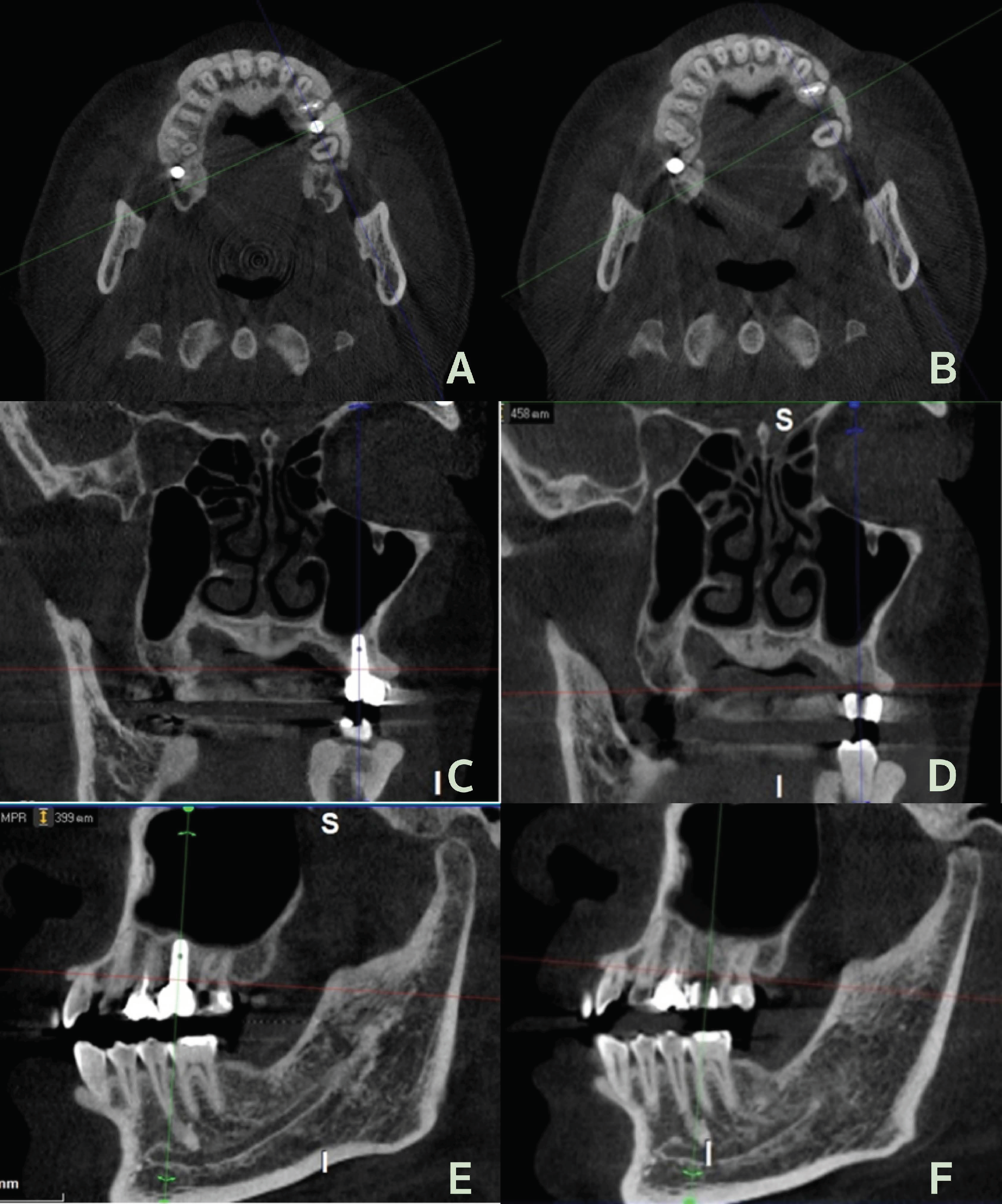

Supplement: Supplementary file 2 — Figure S2. The preoperative (right column) and 6-month postoperative (left column) CBCT images in (A, B) axial view, (C, D) coronal view, and (E, F) sagittal view. The location in the maxilla CBCT images at 6-month postoperation (A, C, E) was recorded and used as the reference location for the preoperative CBCT images (B, D, F). The maxillary anatomical structures surrounding the implant were referenced when aligning the coronal, sagittal, and axial planes to ensure consistent measurement positions. When the postoperative images were produced by CBCT, the position of the mandible may not be in the same location as the preoperative CBCT images. Therefore, the locations in the mandible cannot be used as references. (TIFF 7918 kb) [file 40729_2019_181_MOESM2_ESM.tiff]
